# Primary Atypical Carcinoid Tumour of the Sphenoid Sinus Rostrum

**DOI:** 10.1155/2014/753964

**Published:** 2014-06-26

**Authors:** Kate A. Stephenson, Darlene E. Lubbe

**Affiliations:** Division of Otorhinolaryngology, University of Cape Town, Groote Schuur Hospital, Observatory, H-53 Old Main Building, Cape Town 7925, South Africa

## Abstract

Primary carcinoid tumors of the nasal cavity and sinuses are exceedingly rare. An accurate histopathological diagnosis is crucial to optimal investigation and management. We present a case of a primary atypical carcinoid tumor arising from the sphenoid rostrum without evidence of associated carcinoid syndrome. This rare but important differential diagnosis of a nasal tumor is discussed and important unique management issues are highlighted.

## 1. Introduction

Carcinoid tumours are not well known to otorhinolaryngologists; these neuroendocrine tumours are found almost exclusively below the level of the clavicle. Arising from enterochromaffin cells, they may synthesize a variety of vasoactive substances and hormones. Carcinoids are typically diagnosed in the fifth or sixth decade of life and approximately half of patients may be asymptomatic at diagnosis.

The incidence of carcinoid tumours has been estimated to be 1 to 2 per 100,000 of population. The true incidence is likely to be higher due to the slow-growing, subclinical nature of a large proportion of these lesions. The surveillance, epidemiology, and end result (SEER) program of the National Cancer Institute of the United States of America has analyzed 10,878 carcinoid tumours; 64% were of gastrointestinal origin and 28% originated in the lower respiratory tract [1].

Carcinoids may be classified histologically into “typical” and “atypical” tumours [2]. Typical carcinoids display uniform characteristics without nuclear pleomorphism or mitoses. By comparison, higher mitotic rates, greater nuclear atypia, and necrosis may be seen in atypical carcinoids; these features are associated with more aggressive disease. Both subdivisions of carcinoids may be associated with malignant behavior; local invasion and distant metastases may occur.

We present a case of an atypical carcinoid of the sphenoid sinus rostrum and posterior nasal septum without evidence of carcinoid syndrome. Whilst very few cases of ethmoid and frontal sinus carcinoids have been described, only one carcinoid of the nasal septum has previously been reported [3–5]. The commonest head and neck site of carcinoid tumours is the larynx [6]. To the best of our knowledge, only one other primary atypical carcinoid of the sphenoid sinus has been reported in the English literature; Westerveld et al. described a case of an atypical carcinoid tumour with bony metastatic lesions, which seemed to be associated with multiple endocrine neoplasia type 1 [7]. A single case of a typical carcinoid of the nasopharynx and sphenoid sinus has also been reported [8].

## 2. Case Presentation

A 48-year-old male presented with a 3-month history of mild watery rhinorrhea and pain on nasal blowing, particularly on the left. No epistaxis was reported and there was no history of systemic upset. He was a nonsmoker and had no significant past medical history of note.

**Figure 1:**
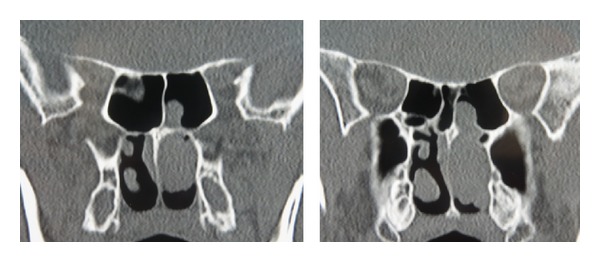
Preoperative CT.

A left-sided nasal polyp emanating from the left sphenoid sinus ostium was identified on initial nasendoscopic examination. High resolution computed tomography (HRCT) showed this soft tissue mass to be situated between the middle turbinate and the nasal septum, extending posteriorly to the level of the posterior choana with evidence of fluid within the left sphenoid sinus (Figure 1). Neither bony nor intracranial involvement was evident. An endoscopic biopsy was performed.

**Figure 2:**
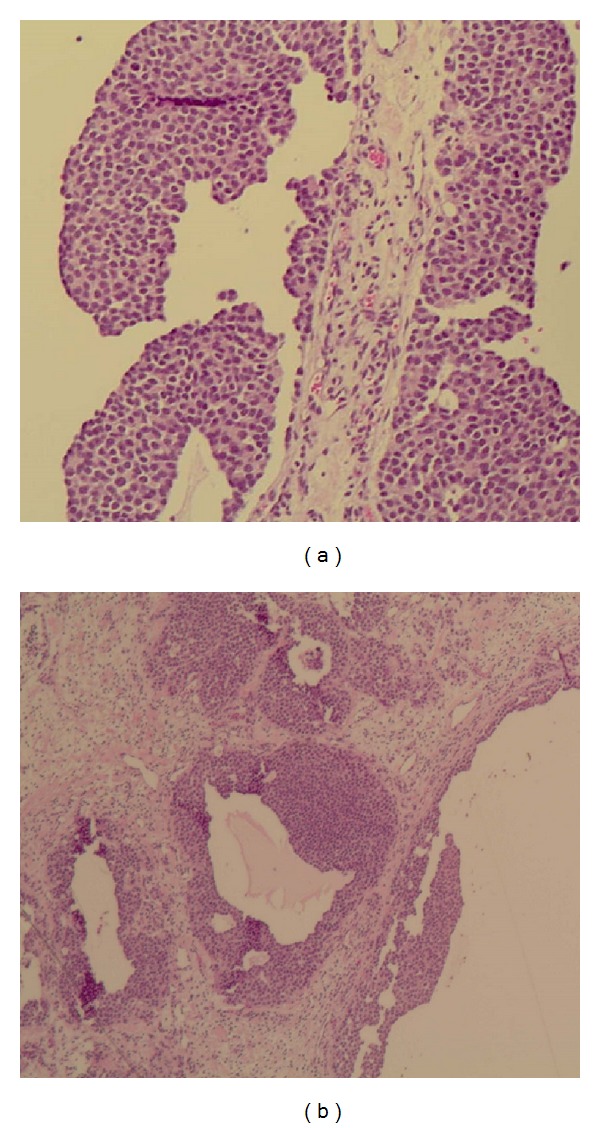
Photomicrograph showing (a) uniform basaloid tumour cells with stippled chromatin and a moderate amount of pale, eosinophilic cytoplasm (H&E; X 10) and (b) cystic changes within the tumour (H&E; X 4).

Histopathological evaluation revealed a polypoid lesion measuring 35 × 20 × 10 mm without atypia of the surface epithelium. Lobules of fairly uniform tumour cells with stippled chromatin and a moderate amount of pale eosinophilic cytoplasm within the lesion were shown on sectioning (Figure 2). Central cystic change, focal apoptosis, and an area of tumour necrosis were also identified.

Immunohistochemical staining demonstrated strong and diffuse uptake of synaptophysin. Synaptophysin is a membrane glycoprotein of neuroendocrine cells and, like chromogranin A, is a valuable specific neuroendocrine marker [9]. Cytokeratin epithelial markers were negative other than those within the surface epithelium. Morphologic features therefore favored a diagnosis of an atypical carcinoid tumour with extension to the excision margin.

Further investigation was undertaken to further evaluate the nature of the tumour; carcinoid syndrome was not evident. Serum serotonin and 24-hour urine 5-hydroxy-indole acetic acid (5-HIAA) levels were within normal limits. Full radiological evaluation and gastrointestinal endoscopy did not reveal distant disease.

Endoscopic resection using navigation technology was then performed. The remaining tumour was found to be pedicled upon the sphenoid rostrum and posterior septum and specifically seemed to arise from the intersinus septum of the sphenoid. An en bloc resection included complete removal of the anterior sphenoid face and removal of all sphenoid sinus mucosa, a posterior septectomy and ipsilateral ethmoidectomy. Histology revealed a small nodular focus of residual tumour with exact pathological correlation with the initial specimen. Tumour involvement of bone was not detected.

A policy of close clinical follow-up was agreed on. A whole body octreotide (radiolabelled indium-111-tegnesium-octreotide) scan performed at an interval of 3 months following surgery showed no uptake in the nasal area and no other identifiable lesions. A positron emission tomography (PET) scan at one year postoperatively did not reveal evidence of disease and repeat lower gastrointestinal endoscopy was normal. At 3 years following surgery the patient is asymptomatic without evidence of local recurrence on detailed nasendoscopic examination.

## 3. Discussion

Once a diagnosis of a sinonasal carcinoid tumour has been established, optimal initial management relies upon appropriate systemic investigation. Presence of a carcinoid tumour must be distinguished from the “carcinoid syndrome.” This describes systemic effects that occur as a result of release of compounds synthesized by the tumour. Symptoms include episodic flushing and diarrhea and the development of respiratory symptoms, such as wheezing. Carcinoid heart disease, characterized by endocardial thickening and valvular fixation predominantly of the right side of the heart, occurs in approximately two-thirds of patients with carcinoid syndrome. This may require surgical management and lead to significant morbidity and mortality.

Carcinoid crisis is a life-threatening form of carcinoid syndrome. Flushing, diarrhea, tachycardia, arrhythmias, hypertension or hypotension, bronchospasm, and altered mental status may occur. Anesthesia, surgery, or chemotherapy administration may precipitate a crisis, thought to be due to the release of compounds secreted by the tumour. Failure to identify either carcinoid syndrome or carcinoid heart disease could therefore result in management-related morbidity and mortality.

Treatment of localized disease is relatively well defined and typically comprises surgical resection, as was performed in our case. Metastatic disease poses a complex therapeutic challenge and several modalities of treatment have been investigated. Carcinoid syndrome may be controlled with somatostatin analogues such as octreotide and in such cases multidisciplinary input is recommended. Beneficial additional use of interferon alpha has been described. Liver metastases may be resected whilst liver transplantation and hepatic arterial embolization have also been explored [10].

Given the relative rarity of primary sinonasal carcinoid disease, little is known of the prognosis. Five-year survival of patients with primary laryngeal carcinoids has been estimated to be 46.7% [11]. An unexpectedly high proportion of metastatic disease from small lesions with invasion limited to mucosa or submucosa has also been identified.

## 4. Conclusion

This case of a sinonasal atypical carcinoid highlights the importance of accurate histopathological evaluation of any polypoid mass within the nasal cavity and paranasal sinuses. A carcinoid tumour is an important differential diagnosis, albeit rare. It is associated with unique management concerns and a need for long-term surveillance with respect to both recurrent local and distant disease.
